# Balloon pulmonary angioplasty vs riociguat in patients with inoperable chronic thromboembolic pulmonary hypertension: A systematic review and meta‐analysis

**DOI:** 10.1002/clc.23212

**Published:** 2019-06-12

**Authors:** Wuwan Wang, Li Wen, Zhengdong Song, Wenhai Shi, Ke Wang, Wei Huang

**Affiliations:** ^1^ Department of Cardiology The First Affiliated Hospital, Chongqing Medical University Chongqing China; ^2^ Department of Cardiology The Sixth People's Hospital of Chengdu Chengdu China; ^3^ Institute of Cardiovascular Diseases of People's Liberation of Army (PLA), Xinqiao Hospital Army Medical University Chongqing China

**Keywords:** balloon pulmonary angioplasty, chronic thromboembolic pulmonary hypertension, efficacy, riociguat, safety

## Abstract

**Backgrounds:**

No previous meta‐analyses have compared the efficacy and safety of BPA with riociguat therapy in inoperable CTEPH patients.

**Methods:**

Relevant published studies were searched in the PubMed, Embase and ClinicalTrial.gov databases.

**Results:**

Twenty‐three clinical trials including 1454 patients (631 underwent BPA; 823 underwent riociguat therapy) were analyzed. BPA was associated with a greater improvement in RAP (mean difference (MD) = −3.53 mmHg, 95% CI: [−4.85, −2.21] vs MD = −1.05 mmHg, 95% CI: [−1.82, −0.29]); mPAP (MD = −15.02 mmHg, 95% CI: [−17.32, −12.71] vs MD = −4.19 mmHg, 95% CI: [−5.58, −2.80]); PVR (standard MD = −1.32 woods, 95% CI: [−1.57, −1.08] vs standard MD = −0.65 woods, 95% CI: [−0.79, −0.50]); NYHA functional class (RR = 6.78, 95% CI: [3.14, 14.64] vs RR = 1.49, 95% CI: [1.07, 2.07]); and 6MWD (MD = 71.66 m, 95% CI: [58.34, 84.99] vs MD = 45.25 m, 95% CI: [36.51, 53.99]) than riociguat treatment. However, the increase in CO was greater with riociguat (MD = 0.78 L/min, 95% CI: [0.61, 0.96]) than with BPA (MD = 0.33 L/min, 95% CI: [0.06, 0.59]). No significant difference in cardiac index (CI) was found between BPA (MD = 0.40 L/min/m^2^, 95% CI: [0.21, 0.58]) and riociguat (MD = 0.40 L/min/m^2^, 95% CI: [0.26, 0.54]). The most common complications of BPA were pulmonary injury (0.3%‐5.6%) and pulmonary edema (0.8%‐28.6%). The most common adverse events of riociguat were headache, dizziness, hypotension and nasopharyngitis.

**Conclusions:**

Our meta‐analysis indicates that BPA might be associated with greater improvements in exercise tolerance and pulmonary hemodynamics except for cardiac output and cardiac index than riociguat therapy. However, both of them were well tolerated.

**Abbreviations**
BNP: brain natriuretic peptide
BPA: balloon pulmonary angioplasty
CO: cardiac output
CTEPH: chronic thromboembolic pulmonary hypertension
mPAP: mean pulmonary artery pressure
NYHA: New York Heart Association
PVR: pulmonary vascular pressure
RAP: right atrium pressure
6MWD: 6‐minute walking distance

## 1 INTRODUCTION

Chronic thromboembolic pulmonary hypertension (CTEPH) is a rare, potentially life‐threatening disease of the pulmonary vasculature.^1^ CTEPH has been proposed to develop when a pulmonary embolism does not resolve and transforms into fibrous tissue that occludes the pulmonary artery.^2^, ^3^, ^4^, ^5^ Patients with untreated CTEPH are at high risk of progressive pulmonary hypertension, right heart failure and death.^5^

For patients with CTEPH, the gold standard treatment is potentially curative pulmonary endarterectomy (PEA).^1^, ^6^ For patients ineligible for PEA or those who have recurrent or persistent pulmonary hypertension after surgery, drug treatment with riociguat is beneficial and recommended by the guideline.^7^ At present, riociguat is the only medicine approved for the treatment of both PAH and inoperable, persistent or recurrent CTEPH.^8^, ^9^

Balloon pulmonary angioplasty (BPA) is an emerging option that promises hemodynamic and functional benefits for inoperable patients.^10^ BPA relies on the use of telescoping catheters placed in a central vein, through which wires and balloons are guided to mechanically disrupt chronic clot material and relieve pulmonary vascular obstruction. Recently, additional groups have reported that BPA improved symptoms and hemodynamic parameters in patients with peripheral‐type CTEPH.^11^ In addition, repeated PEA is not a feasible way due to high perioperative risk. Therefore, BPA appears to be an alternative and less invasive technique and may address some of the limitations of PEA.^12^, ^13^, ^14^ In the past 5 years, BPA has been widely performed worldwide. BPA currently carries a class IIb recommendation for the treatment of inoperable CTEPH according to the most recent European guidelines^15^ and may be considered in patients who are technically inoperable or carry an unfavorable risk during surgery.

One meta‐analysis compared the efficacy of medical therapy, which included pulmonary vasodilators, against BPA in patients with inoperable CTEPH.^16^ In this meta‐analysis, six studies on BPA and 15 studies on medical therapy, including various pulmonary vasodilators with heterogeneity, were pooled. The conclusion showed high‐quality evidence on the use of pulmonary vasodilators while only moderate‐quality evidence on BPA in improving both hemodynamics and exercise capacity. However, no previous meta‐analyses have evaluated and directly compared the efficacy and safety of BPA with those of riociguat therapy. Additional clinical trials on BPA have recently been published. Therefore, the aim of this new meta‐analysis was to evaluate and compare the efficacy and safety of BPA with riociguat therapy, including 11 recent clinical trials on BPA, in inoperable CTEPH patients.

## 2 METHODS

### 2.1 Search strategy

We performed a review of the literature and a meta‐analysis of studies that compared the efficacy and safety of BPA against those of riociguat therapy in inoperable CTEPH patients; hemodynamic parameters of right heart catheterization (RHC), 6‐minute walking distance (6MWD) and New York Heart Association (NYHA) functional class were evaluated. Relevant studies were identified by searching the PubMed and Embase databases and ClinicalTrial.gov using the following search terms: (“chronic thromboembolic pulmonary hypertension” OR “chronic pulmonary embolism”) AND (“percutaneous transluminal pulmonary angioplasty” OR “BPA” OR “balloon pulmonary angioplasty” OR “riociguat”). In addition, the references of all retrieved literatures were reviewed for further identification of potentially relevant studies. This meta‐analysis was performed according to the Preferred Reporting Items for Systematic Reviews and Meta‐Analyses (PRISMA) statement.^17^ All studies included in this meta‐analysis were published from January 2001 to September 2017.

### 2.2 Selection criteria

Studies were identified for inclusion by screening “titles/abstracts and full texts” if they met all of the following criteria: (i) the subjects were diagnosed with inoperable CTEPH by demonstration of organized pulmonary thromboembolism using contrast‐enhanced lung computed tomography, perfusion lung scintigraphy, and pulmonary angiography, excluding collagen vascular disease, pulmonary disease, left heart abnormality, and other systemic diseases by blood tests, pulmonary function tests, and echocardiography. Patients with residual or recurrent pulmonary hypertension after pulmonary endarterectomy were enrolled. (ii) Group 1 included patients who underwent BPA, and group 2 included patients who were administered riociguat as the first prescribed treatment or add‐on medication; (iii) all included studies were retrospective or prospective clinical studies; (iv) the primary exposure investigated had to include hemodynamic parameters of RHC, NYHA functional assessments, 6MWD and brain natriuretic peptide (BNP), and complications after BPA were also assessed; Pulmonary edema was defined as X‐ray opacity in the lung segment treated with BPA on the day or next day of the procedure.^18^ Pulmonary vascular injury was commonly caused by wire perfusion, resulting in parenchymal bleeding with or without hemoptysis.^19^ and (v) all patients were diagnosed as inoperable by experienced surgeons due to the location of thrombi and surgical accessibility, age, and comorbidities.

### 2.3 Data extraction

The characteristics of the included clinical trials were independently extracted by two authors (W.W. and W.L.).

### 2.4 Assessment of study quality

The Newcastle‐Ottawa Scale (NOS)^20^ for assessing the quality of nonrandomized studies in meta‐analyses was used to assess the risk of bias, which consisted of the following three aspects: selection, comparability, and outcome. All included studies were prospective or retrospective single‐arm studies. Quality assessment was independently conducted by two authors (W.W and W.L.); their results were compared, and if a consensus could not be reached, a third person (S.Z.) intervened.

### 2.5 Statistical analyses

Pooled treatment effects, including NYHA functional class, 6MWD, BNP and hemodynamic parameters of RHC, were estimated using STATA software (Version 12). A *P*‐value less than .05 for any statistical test was regarded as statistically significant. For continuous data, the inverse variance statistical method was used to measure the effect of the mean difference in each outcome. Categorical variables were compared using Chi‐squared or Fisher's exact tests. We used Cochran's *χ* ^2^‐based Q test and the I‐squared test to assess interstudy heterogeneity.^21^ If there was no significant heterogeneity (defined as *P >* .10 or *I* ^2^ < 50%), the pooled outcomes were determined with the fixed effects model (Mantel‐Haenszel). Conversely, the random effects model (DerSimonian and Laird) was used when significant heterogeneity was found.^22^ In addition, a sensitivity analysis was performed to determine the effects of individual trials on the overall pooled results.

Furthermore, potential publication bias was considered using Begg's rank correlation test^23^ and Egger's linear regression test.^24^ Funnel plots were employed to assess potential publication bias.

## 3 RESULTS

### 3.1 Literature search

**Figure 1:**
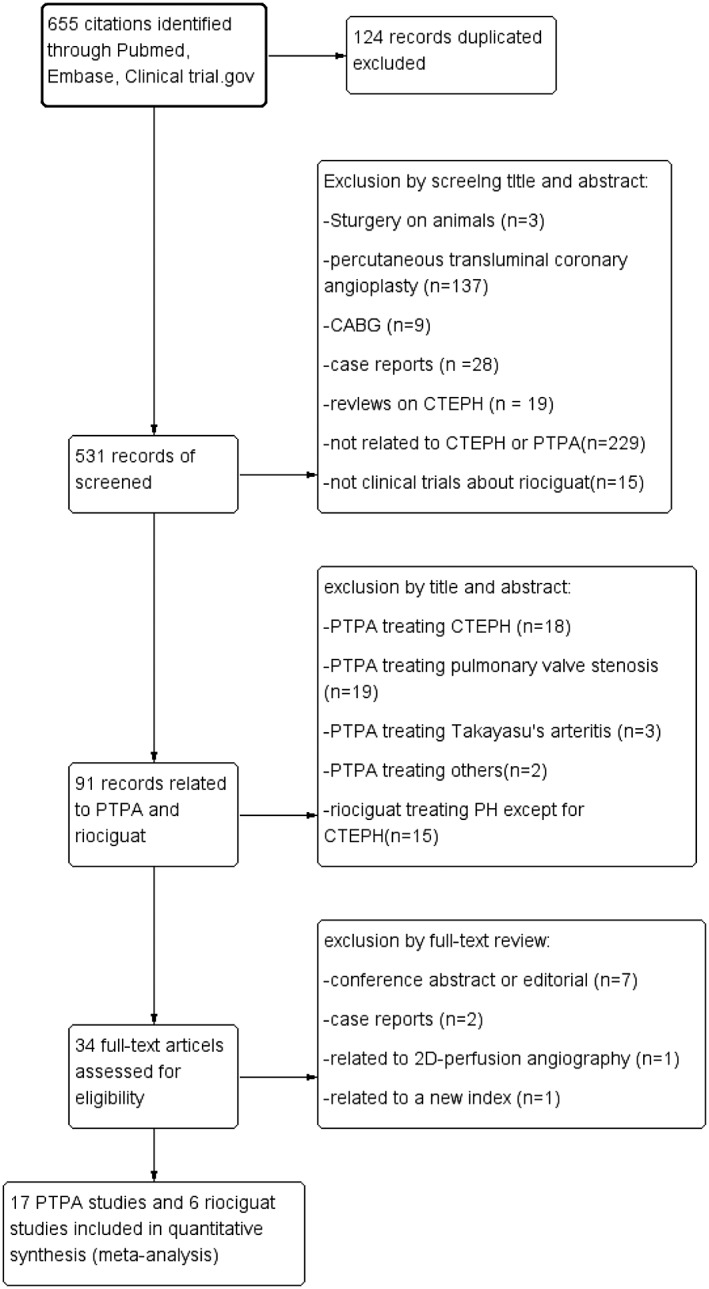
Flow diagram of the study selection process. The flow diagram shows the literature search for relevant studies on the effect and safety of BPA vs riociguat in patients with inoperable CTEPH

A total of 655 citations were identified, and 124 duplicates were removed, leaving 531 studies for screening. After reviewing the titles and abstracts, 440 publications were excluded because they did not report on BPA performed in humans or were not clinical trials about riociguat therapy. The 34 studies on inoperable CTEPH underwent full‐text review; seven studies were excluded because the articles were conference abstracts or editorials, and two articles were excluded because they were related to 2D‐perfusion angiography and a new index. In addition, two case reports were excluded. After reviewing the remaining studies, 17^14^, ^18^, ^19^, ^25^, ^26^, ^27^, ^28^, ^29^, ^30^, ^31^, ^32^, ^33^, ^34^, ^35^, ^36^, ^37^, ^38^ studies of BPA and 6^7^, ^39^, ^40^, ^41^, ^42^, ^43^ studies of riociguat therapy met the inclusion criteria and were included in the pooled analysis (Figure 1).

### 3.2 Eligible studies

Table 1 summarized the main characteristics of the 23 included studies. A total of 1454 patients, of whom 631 underwent BPA treatment, and 823 underwent riociguat treatment, were included from January 2001 to September 2017.

**Table 1:** Characteristics of the trials included in the meta‐analysis

Studies	Subgroup	Sample size	BPA sessions	Patient age (years)	Time of follow‐up	Baseline differences of outcomes	Number of patients with pretreatment of medication	Number of patients under medication at follow‐up time	Complications
Feinstein et al^25^	BPA	18	107	52 ± 12	13 ± 22 months	NYHA class (class I, II 0%), mPAP (43 mmHg)	NR	NR	One patient died from recurrent aspiration pneumonia, reperfusion pulmonary edema (30%)
Sugimura et al^26^	BPA	12	NR	58 ± 13	12 months	NYHA class (class I, II 33.3%), mPAP (47.8 mmHg), RAP (7.3 mmHg), Cl (2.2 L/min/m^2^), PVR (12.1 woods), 6MWD (350 m), BNP (335 pg/mL)	11 (Sildenafil); 5 (Beraprost); 7 (Epoprostenol); 5 (Bosentan)	11 (Sildenafil); 11 (Beraprost); 0 (Epoprostenol) 5 (Bosentan)	Hemoptysis (50%)
Mizoguchi et al^27^	BPA	68	255	62.2 ± 11.9	2.2 ± 1.4 years	NYHA class (class I, II 0%), sPAP, mPAP (45.4 mmHg), RAP (8.1 mmHg), Cl (2.2 L/min/m^2^), PVR (11.8 woods), 6MWD (296 m), BNP (335 pg/mL), oxygen inhalation	52% (ERA); 40% (PDE5 inhibitor); 4 (epoprostenol)；	37% (ERA); 28% (PDE5 inhibitor); 0 (epoprostenol)	Reperfusion pulmonary edema (17.8%), pulmonary artery injury (perforation) (2%)
Andreassen et al^28^	BPA	20	73	60.0 ± 10.0	51.0 ± 30.0 months	NYHA class (class I, II 15%), mPAP (45 mmHg), CO (4.9 L/min)	2 (Sildenafil) (And none received any specific medication for PH at follow‐up evaluation.)	None.	Two patients died, reperfusion pulmonary edema (9.6%)
Fukui et al^29^	BPA	20	3.2 ± 0.9 per patient	67.0 ± 9.0	NR	mPAP (39.4 mmHg), PVR (11.1 woods), CI (2.2 L/min/m^2^), 6MWD (361 m), BNP (175 pg/mL)	6 (ERA); 13 (Oral prostacyclin analogue); 4 (PDE5 inhibitor); 6 (combination therapy)	NR	No death or major complication
Inami et al^30^ (JACC)	BPA	103	350	65 ± 14.1	14.0 ± 10.6 months	mPAP (41 mmHg), RAP (5 mmHg), Cl (2.5 L/min/m^2^), PVR (8.7 woods), 6MWD (360 m), BNP (94.5 pg/mL)	NR	NR	Reperfusion pulmonary edema (16.6%)
Shimura et al^14^	BPA	9	44	55.1 ± 12.4	1.9 ± 1.5 years	NYHA class (class I, II 33.3%), mPAP (43 mmHg), CO (3.9 L/min), PVR (8.1 woods), BNP (49.2 pg/mL)	NR	NR	Reperfusion pulmonary edema (2.3%), vessel injury (0.6%)
Kurzyna et al^31^	BPA	20	37	NR	NR	NYHA class (class I, II 5%), mPAP (58 mmHg), PVR (11.7 woods)	NR	NR	NR
Velázquez Martín^47^	BPA	7	20	61	6 ± 12 months	mPAP (56 mmHg), PVR (11.8 woods), CI (2.3 L/min/m^2^)	NR	NR	Pulmonary edema (28.6%)
Roik et al^33^	BPA	9	27	76 ± 18	5 ± 6 months	NYHA class (class I, II 0%), mPAP (40 mHg), PVR (9.1 woods), CO (3.8 L/min), CI (2.2 L/min/m^2^), 6MWD (304 m)	6 (Sildenafil)	NR	Reperfusion pulmonary edema (22.2%), vessel injury (perforation) (0%)
Koike et al^35^	BPA	8	16	70.8 ± 8.6	NR	mPAP (30.4 mmHg), Cl (2.7 L/min/m^2^), CO (4.0 L/min), PVR (6 woods), 6MWD (332 m)	NR	NR	NR
Kimura et al^36^	BPA	66	446	63.2 ± 13.2	5.1 ± 3.74 months	RAP (6.6 mmHg), mPAP (39.2 mmHg), PVR (9.5 woods), BNP (238 pg/mL)	7 (Riociguat); 35 (PDE5 inhibitor); 27 (ERA); 19(Prostacyclin analogue)	NR	Hemosputum (6.1%)
Ogo et al^37^	BPA	80	385	68 ± 13.3	1 year	mPAP (42 mmHg), RAP (3.9 mmHg), PVR (11 woods), C I (2.3 L/min/m^2^), 6MWD (372 m), BNP (227 pg/mL)	4 (Riociguat); 26 (ERA); 33(prostacyclin analogue); 20 (PDE5 inhibitor)	NR	Reperfusion pulmonary edema (4.7%), vessel injury (perforation) (0.3%)
Yamasaki et al^38^	BPA	20	2.7 per patient	61.9 ± 10.6	88 ± 50 days	mPAP (42.6 mmHg), PVR (8 woods), CI (3.1 L/min/m^2^), 6MWD (391 m), BNP (66.5 pg/mL)	9 (Riociguat); 10 (PDE5 inhibitor); 9 (ERA); 8(Prostacyclin analogue)	NR	NR
Olsson et al^19^	BPA	56	266	65 ± 14.1	6 months	NYHA class (class I, II 16.1%), RAP (8 mmHg), mPAP (40 mmHg), PVR (7.4 woods), CO (4.4 L/min), CI (2.4 L/min/m^2^), 6MWD (358 m)	8 (Riociguat); 33 (PDE5 inhibitor); 10 (ERA); 1 (Prostacyclin)	NR	Reperfusion pulmonary edema (0.8%), vessel injury (0.6%)
Kurzyna et al.^46^	BPA	31	117	58.6 ± 17.9	12.5 months	NYHA class (class I, II 3.2%), RAP (10.4 mmHg), mPAP (50.7 mmHg), PVR (10.3 woods), CO (4.2 L/min), CI (2.3 L/min/m^2^), 6MWD (306 m)	NR	NR	Hemoptysis (10%), vessel injury (13%)
Aoki et al^34^	BPA	84	424	65 ± 14	31 ± 5.25 months	mPAP (38 mmHg), PVR (7.3 woods), 6MWD (380 m), BNP (55.8 pg/mL)	13 (Riociguat); 56 (PDE5 inhibitor); 13 (ERA); 34 (Prostacyclin); 12 (Epoprostenol)	22(Any vasodilators); 0 (Epoprostenol)	pulmonary edema (1%), Haemoptysis (14%), Pulmonary arterial dissection (7%)
Ghofrani et al^39^	Riociguat	41	N/A	44 [38‐51]	3 months	mPAP (44 mmHg), PVR (8.6 woods), CI (2.3 L/min/m^2^), 6MWD (390 m)	N/A	N/A	Dyspepsia (24%), headache (16%), hypotension (15%), peripheral edema (12%)
Yamamoto et al^41^	Riociguat	23	N/A	65.7 ± 10.1	6‐12 months	NYHA class (class I, II 87%), mPAP (38.8 mmHg), PVR (7.24 woods), CI (2.9 L/min/m^2^), 6MWD (376 m), BNP (147.3 pg/mL)	N/A	N/A	Headache (30.4%), hypotension (17.4%), dizziness (8.7%), hemoptysis (8.7%)
McLaughlin et al^43^	Riociguat	258	N/A	63.9 ± 12.5	48 weeks	6MWD (374 m), NYHA class (class I, II 39%)	N/A	N/A	Dyspepsia (20%), dizziness (19%), headache (18%), peripheral edema (18%), diarrhea (15%), nausea (13%)
Ghofrani et al^40^	Riociguat	173	N/A	59 ± 14	16 weeks	NYHA class (class I, II 33.5%), mPAP (45 mmHg), PVR (9.9 woods), RAP (9 mmHg), CO (4 L/min), 6MWD (342 m)	N/A	N/A	Headache (25%), dizziness (23%), dyspepsia (18%), peripheral edema (16%)
Simonneau et al^7^	Riociguat	155	N/A	59 ± 14	1 year	6MWD (351 m), NYHA class (class I, II 32%)	N/A	N/A	Nasopharyngitis (24%), dizziness (19%), peripheral edema (15%), diarrhea (14%)
Kim et al^42^	Riociguat	173	N/A	59 ± 14	16 weeks	6MWD (335/360 m), mPAP (47/40 mmHg), PVR (10.8/7.3 woods), RAP (8.3/9.3 mmHg), CO (4.1/4.2 L/min), CI (2.3 L/min/m^2^)	N/A	N/A	Dizziness (22%), headache (21%), dyspepsia (11%), peripheral edema (14%)

Abbreviations: BNP, brain natriuretic peptide; CO, cardiac output; CI, cardiac index; ERA, Endothelin receptor antagonist; mPAP, mean pulmonary artery pressure; N/A, not applicable; NR, not reported; NYHA, New York Heart Association; PDE5 inhibitor, Phosphodiesterase type‐5 inhibitor; PH, pulmonary hypertension; PVR, pulmonary vascular pressure; RAP, right atrium pressure; and 6MWD, six‐minute walking distance.

### 3.3 Methodological quality assessment

The quality of each included study was assessed using NOS. Thirteen studies received eight stars, nine studies^7^, ^30^, ^35^, ^37^, ^39^, ^40^, ^41^, ^42^, ^43^ received nine stars, and one study^31^ received seven stars.

### 3.4 Overall analysis of efficacy endpoints

#### 3.4.1 Hemodynamic parameters

**Figure 2:**
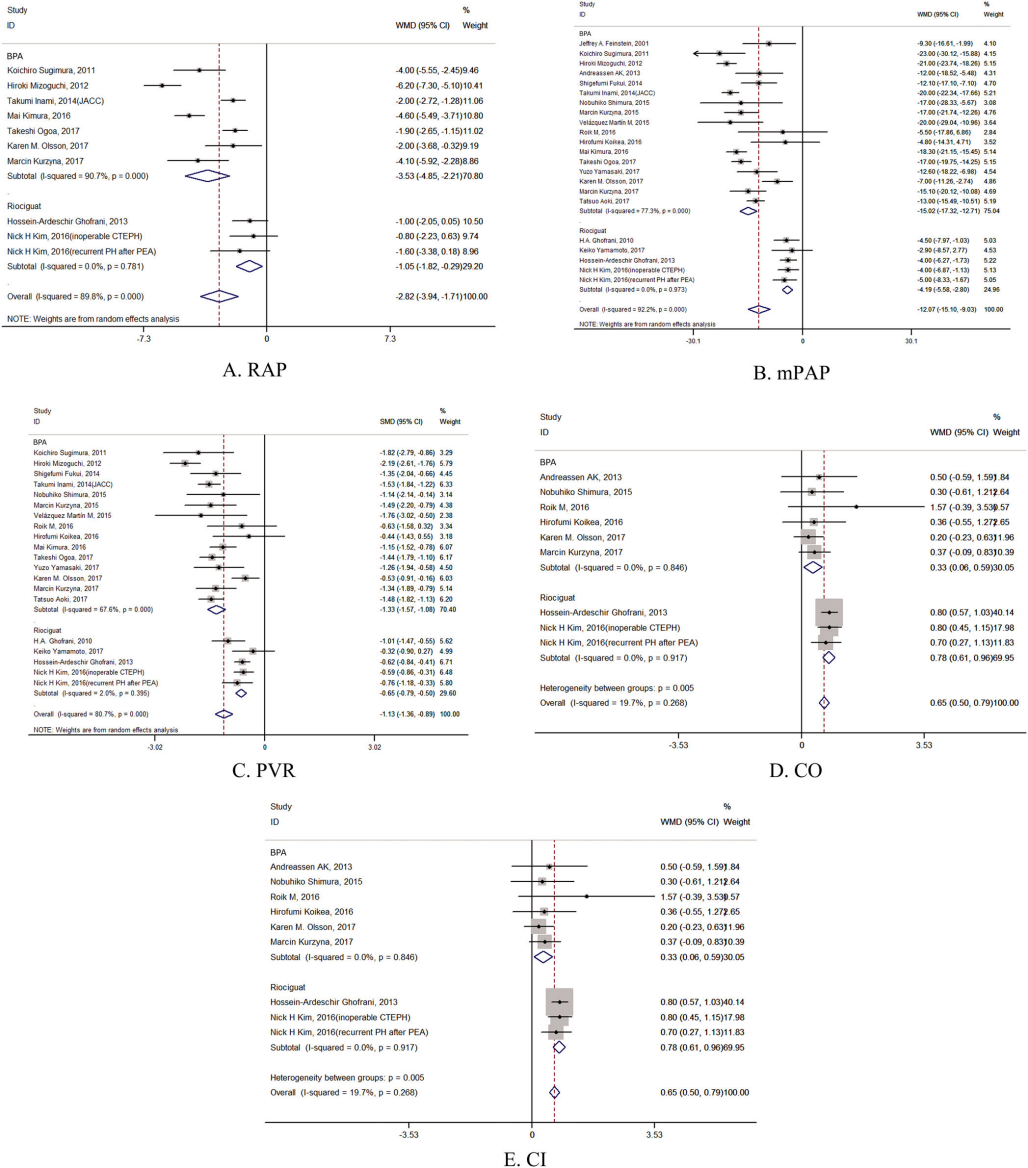
Forest plots of the clinical outcomes of hemodynamic parameters. Pooled differences in the means of (A) right atrium pressure (RAP), (B) mean pulmonary arterial pressure (mPAP), (C) pulmonary vascular resistance (PVR), (D) cardiac output (CO) and (E) cardiac index after balloon pulmonary angioplasty (BPA). CI, confidence interval. [Correction added on 02‐July 2019, after first online publication: Figures 2A and 2B have been replaced with updated figures that correct spacing problems in the original version of the figures.]

The random effects model was utilized for the analysis. Regarding hemodynamic parameters, RAP was significantly reduced after BPA (mean difference = −3.5 mmHg, 95% CI: [−4.85, −2.21], *P* = .000) with severe heterogeneity (*I* ^2^ = 90.7%) (Figure 2A), whereas RAP was also significantly reduced after administration of riociguat (mean difference = −1.1 mmHg, 95% CI: [−1.82, −0.29], *P* = .007) without heterogeneity. As shown in Figure 2A, the pooled improvement of RAP in the BPA group was greater than that in the riociguat therapy group.

BPA also significantly reduced mean pulmonary artery pressure (mPAP) (mean difference = −15.0 mmHg, 95% CI: [−17.32, −12.71], *P* = .000) with severe heterogeneity (*I* ^2^ = 77.3%) under the random effects model (Figure 2B). Among patients who underwent riociguat therapy, mPAP was significantly reduced without heterogeneity (mean difference = −4.2 mmHg, 95% CI: [−5.58, −2.80], *P* = .000). However, mPAP was less improved with riociguat therapy than with BPA.

Pulmonary vascular resistance (PVR) was significantly decreased after BPA (standard mean difference = −1.3 woods, 95% CI: [−1.57, −1.08], *P* = .000 with severe heterogeneity (*I* ^2^ = 67.6%) (Figure 2C). The improvement in PVR in the riociguat therapy group was less than in the BPA group (standard mean difference = −0.7 woods, 95% CI: [−0.79, −0.50], *P* = .000) with mild heterogeneity (*I* ^2^ = 2.0%).

In addition, BPA significantly increased cardiac output (CO) (mean difference = 0.3 L/min, 95% CI: [0.06, 0.59], *P* = .018) without heterogeneity (*I* ^2^ = 0.0%) (Figure 2D). However, CO was significantly increased with riociguat therapy, and the improvement in CO was greater with riociguat therapy than with BPA (mean difference = 0.8 L/min, 95% CI: [0.61, 0.96], *P* = .000) without heterogeneity (*I* ^2^ = .0%).

The cardiac index of inoperable CTEPH patients was also significantly increased similarly in both groups (mean difference = 0.4 L/min/m^2^, 95% CI: [0.21, 0.58], *P* = .000) with severe heterogeneity (*I* ^2^ = 77.5%) (Figure 2E). No significant difference was found in CI between the riociguat therapy and BPA cohorts (mean difference = 0.4 L/min/m^2^, 95% CI: [0.26, 0.54], *P* = .000).

#### 3.4.2 Functional capacity

**Figure 3:**
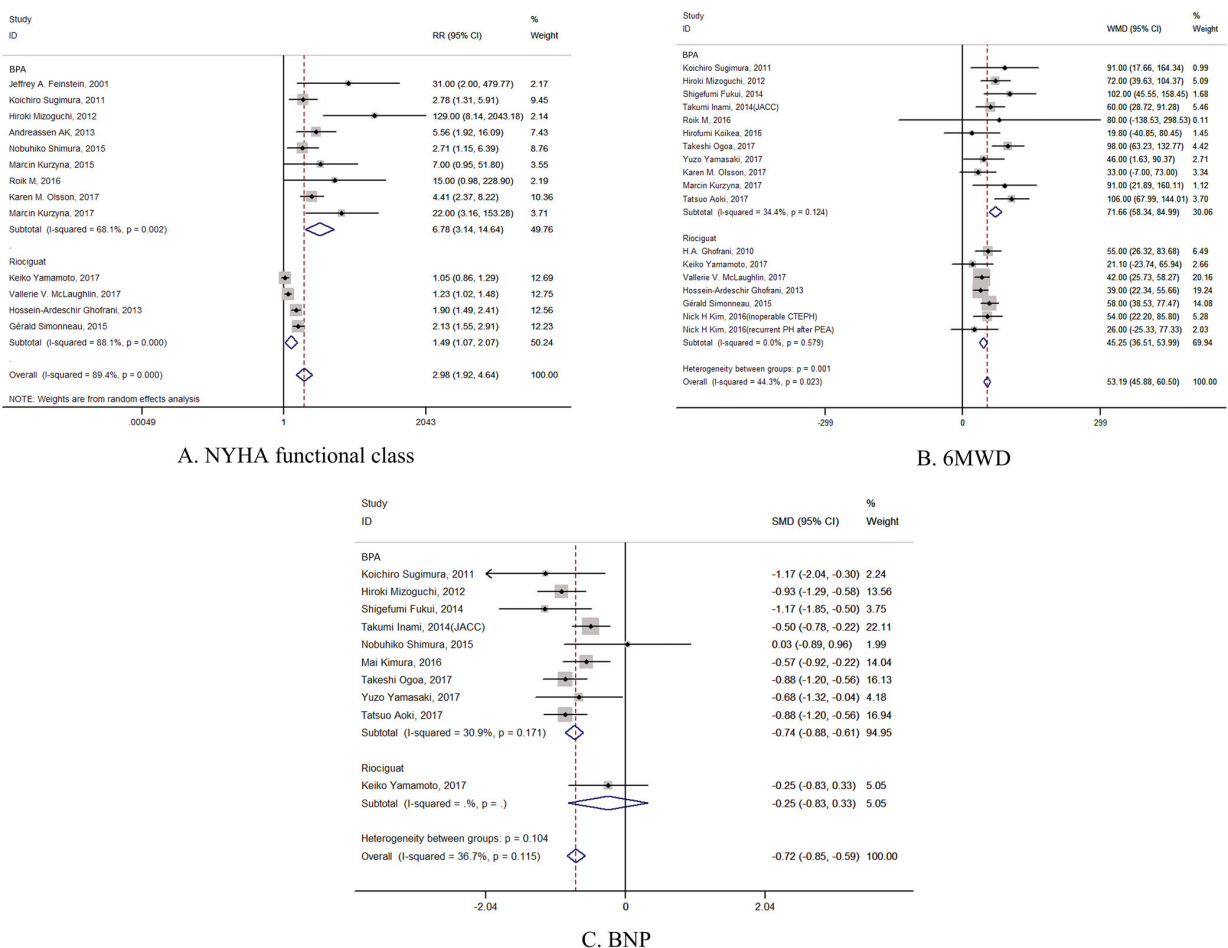
Forest plots of the clinical outcomes of exercise tolerance. Pooled differences in the means of (A) NYHA functional class, (B) 6‐minute walking distance (6MWD), and (C) brain natriuretic peptide (BNP) after balloon pulmonary angioplasty (BPA). CI, confidence interval

BPA treatment significantly improved the NYHA class in the inoperable CTEPH patients (RR = 6.8, 95% CI: [3.14, 14.64], *P* = .000) (Figure 3A). The random effects model was used in the analysis of NYHA across the studies because it was statistically heterogeneous (*I* ^2^ = 68.1% in BPA group and *I* ^2^ = 88.1% in riociguat group). The likelihood of improvement in NYHA functional class in the riociguat group was less than in the BPA group (RR = 1.5, 95% CI: [1.07, 2.07], *P* = .018).

The treatment of BPA in the inoperable CTEPH patients led to significant improvement in the 6MWD (mean difference = 71.7 m, 95% CI: [58.34, 84.99], *P* = .000) with mild heterogeneity (*I* ^2^ = 34.4%) (Figure 3B). Therefore, the fixed effects model was used. The changes were also greater in patients with BPA than those with riociguat therapy (mean difference = 45.3 m, 95% CI: [36.51, 53.99], *P* = .000) without any heterogeneity.

Furthermore, the BNP levels before and after BPA were evaluated. These findings indicated that BPA significantly reduced BNP levels in inoperable CTEPH patients (standard mean difference = −0.7 pg/mL, 95% CI: [−0.88, −0.61], *P* = .000) with mild heterogeneity (*I* ^2^ = 30.9%) (Figure 3C). However, only one study reported the BNP level in CTEPH patients with riociguat therapy (standard mean difference = −0.3 pg/mL, 95% CI: [−0.83, −0.33]). Thus, we did not compare it with BPA vs riociguat.

#### 3.4.3 Complications

Complication rates were reported for the 17 studies. After BPA, the most common symptom among the CTEPH patients was hemoptysis, which is usually caused by wire perforation.^10^ Moreover, the most common complications were pulmonary edema and pulmonary injury. Among the included studies that reported these complications, the reperfusion pulmonary edema rate ranged from 0.8% to 28.6%, and the pulmonary injury rate ranged from 0.3% to 5.6%. Only one study reported that one participant had died due to pulmonary artery wiring perforation after the procedure. In terms of riociguat treatment among the inoperable CTEPH patients, the most common adverse events observed within the six included studies were dyspepsia, headache, dizziness, hypotension and nasopharyngitis, with an incidence rate of less than 30%. Overall, the BPA and riociguat treatments were both well tolerated.

#### 3.4.4 Sensitivity analysis and publication bias

We performed sensitivity analyses to identify the potential heterogeneity in the efficacy of BPA in inoperable CTEPH patients. For PVR with severe heterogeneity, with the omission of one study,^27^ the pooled improvement changed from (standard mean difference = −1.3, 95% CI: [−1.57, −1.08]) with *I* ^2^ = 67.6% to −1.3 (95% CI: [−1.46, −1.03]) with *I* ^2^ = 51.8%. Moreover, for CI assessment, when one study^27^ was removed, the heterogeneity changed from *I* ^2^ = 77.5% to *I* ^2^ = 21.3%, accompanied by a resulting change from mean difference = 0.4, 95% CI: [0.21, 0.58]) to (mean difference = 0.3, 95% CI: [0.21, 0.42]). Thus, the heterogeneity was attributed to these studies. In the evaluation of RAP, when four studies were removed,^27^, ^30^, ^36^, ^37^ the heterogeneity changed from *I* ^2^ = 90.7% to *I* ^2^ = 47.6%, when the result changed from mean difference = −3.5, 95% CI: [−4.85, −2.21]) to −3.4 (95% CI: [−4.70, −2.02]). However, we still retained them. Thus, we chose a random effects model to estimate the pooled outcomes.

Begg's rank correlation test and Egger's linear regression test were performed to assess whether there was publication bias. As the results showed, publication biases of the included studies were found in the evaluation of NYHA functional class (*P*_Begg_ = 0.200, *P*_Egger_ = 0.001) and BNP level (*P*_Begg_ = 0.474, *P*_Egger_ = 0.008). There was no publication bias found in other outcomes, including hemodynamic parameters and cardiac function.

## 4 DISCUSSION

A previous meta‐analysis^16^ comparing pulmonary vasodilators with BPA showed that there is high‐quality evidence supporting the use of pulmonary vasodilators in improving hemodynamics in patients with inoperable CTEPH, with weaker evidence supporting its benefit for improving exercise capacity. And only moderate‐quality evidence was found from observational studies supporting the efficacy of BPA in improving both hemodynamics and exercise capacity. These results were pooled from 6 studies of BPA and 15 studies of medical therapy. However, the medications utilized were heterogeneous, containing bosentan, sildenafil, beraprost, riociguat, intravenous epoprostenol and subcutaneous treprostenil. Riociguat is currently the only medical therapy approved for the treatment of CTEPH and has been shown to improve hemodynamics and exercise capacity (class I recommendation, level of evidence B).^1^ Phosphodiesterase (PDE)‐5 inhibitors, endothelin receptor antagonists (ERAs) and prostanoids have not been not approved for CTEPH due to a lack of evidence from a reliable randomized study, and they have not been submitted for approval to regulatory bodies (class IIb recommendation, level of evidence B).^1^ In MERIT‐1 (Macitentan for the treatment of inoperable chronic thromboembolic pulmonary hypertension),^44^ a multicenter, phase 2, randomized, double‐blind, placebo‐controlled study, macitentan significantly improved PVR in patients with inoperable CTEPH and was well tolerated. In the present study, data supporting the role of BPA in inoperable CTEPH were limited to observational studies due to the lack of randomized control trial (RCT) data available for BPA. The results must be interpreted with caution and should be further confirmed with multicenter RCTs. An ongoing RACE trial (Riociguat vs Balloon Pulmonary Angioplasty in Non‐operable Chronic thrombo‐embolic Pulmonary Hypertension; NCT02634203) will address the comparative benefit of riociguat vs BPA for inoperable CTEPH, and its results are eagerly awaited. Our pooled results showed that BPA might be associated with greater improvements in exercise tolerance (6MWD, NYHA functional class) and pulmonary hemodynamics (mPAP, PVR and RAP) but not CO and cardiac index compared to riociguat therapy. Four studies with totally 184 patients showed decreased percentage of patients relying on pulmonary vasodilators at follow‐up time after BPA performance.^18^, ^26^, ^27^, ^28^ One study reported that the number of patients who required oxygen therapy on admission was significantly decreased from 83% to 49% after BPA therapy.^18^ The possible reasons may be as follows: Firstly, the identification of the location and characters of thromboembolic lesions is important in patient management because it determines the optimal therapy choice. Lesions in the proximal main, lobar and segmental arteries^6^, ^45^and, in some cases, distally located midsegmental and subsegmental branches^45^ can be surgically removed by pulmonary endarterectomy. At experienced centers, segmental and subsegmental resection can be performed with excellent effects. Distal lesions that are not deemed accessible by pulmonary endarterectomy may be amenable to BPA.^37^ For vessels of 0.1‐0.5 mm in diameter (microvasculature), medication might be the only choice. We consider that different target ranges of BPA vs riociguat contributed to the effect outcomes shown in our analysis. BPA was shown to be associated with greater improvement in exercise tolerance and pulmonary hemodynamic parameters due to the greater targeted ranges of pulmonary arteries, thereby leading to increased revascularization. Riociguat acts only on the microvasculature of distal pulmonary microarteries. Secondly, the present study showed that CO increased more with riociguat than with BPA whereas increases in cardiac index did not differ. Riociguat decreases not only PVR but also systemic vessel resistance. Hypotension is well known side effect of riociguat.^40^ Greater increase of CO with riociguat might be caused by left ventricular afterload reduction through decrease of systemic vessel resistance.^39^ Additionally, we considered that body surface area may manifest a differential change. Besides, different studies were included in the different analyses of CO and CI, which might also be another reason for the difference. The disparity among the range of complications might be largely due to studies being conducted at different times from 2001 to 2017. The complication rates were significantly decreased over time, possibly due to improvements in the BPA procedure.

Due to the heterogeneity observed among the included studies, pooled estimates were calculated using different effects models. For RAP, PVR and cardiac index, in which severe heterogeneity was found, sensitivity analysis identified the source of heterogeneity from each contributing study. In addition, publication bias was found during the assessment. The following reasons might have contributed to the observed heterogeneity: i) the follow‐up year (2.2 ± 1.4 years) of one study^27^ was notably different from the time period reported in other studies, which might have affected the measured outcomes. With a longer follow‐up time, a more dramatic change may be seen in the hemodynamics and exercise tolerance.^27^ ii) The procedure was relatively different in two studies.^27^, ^30^ According to the statistical data, the mean number of sessions per patient enrolled in each study was approximately 2‐3 sessions. However, 2‐8 sessions per patient and 1‐14 vessels dilated per session was reported in one study,^27^ demonstrating that more dilated vessels might facilitate hemodynamic improvement and exercise tolerance. Furthermore, the Pulmonary Edema Predictive Scoring Index+Pressure‐Wire‐Guided technique (PEPSI+PWG) was used in one study.^30^ The NYHA functional class also showed severe heterogeneity, which was largely attributed to one study^27^ in the sensitivity analysis. The random effects model was chosen, assuming that the underlying true effects differed between studies. Formal statistical tests suggested that there was evidence of publication bias with asymmetric funnel plots and Begg's and Egger's tests.

There were some limitations that should be noted. First, all included studies were nonrandomized observational studies. Although we aimed to avoid bias through various means, due to the limitations of the meta‐analysis itself, some bias still existed. However, the extent of bias was within the acceptable range. Second, in some of the included studies the medical pretreatment might influence the outcome of BPA. In order to stabilize the condition, pretreated with pulmonary vasodilators before BPA may be unavoidable in some patients. Thus, we indicated those patients' clinical characters in Table 1. Third, there should be steep learning curve to perform complete BPA unlike in prescribing riociguat. Treatment goal of BPA would also be changed depending on the operators' experience. Therefore, selecting only initial experience of BPA or only latest experience of BPA in each institute might influence the outcome of this study. Fourth, the definition of inoperable CTEPH remains subjective and is highly dependent on the assessment of the local multidisciplinary CTEPH team based on their surgical experience. This issue is of relevance because patients enrolled in the current studies may have been considered to have potentially operable indications if evaluated by another more experienced CTEPH team. Thus, the present systematic review and meta‐analysis is limited by the potential bias introduced by the lack of a standard definition of inoperable CTEPH. Therefore, our findings should be considered carefully and confirmed with further multicenter RCTs and long‐term follow‐up studies.

## 5 CONCLUSION

Our meta‐analysis indicates that both BPA and riociguat improve pulmonary hemodynamic parameters and exercise tolerance. BPA might be associated with greater improvements in exercise tolerance (6MWD, NYHA functional class) and pulmonary hemodynamics (mPAP, PVR and RAP) but not CO and cardiac index compared to riociguat therapy. The most common complications of BPA were pulmonary edema and pulmonary injury. For riociguat, the most common adverse events were dyspepsia, headache, dizziness, hypotension and nasopharyngitis. Overall, both BPA and riociguat were well tolerated. However, our findings need to be confirmed with further multicenter randomized control trials (RCTs) and prospective observational studies.

## CONFLICT OF INTEREST

The authors declare no potential conflict of interests.
