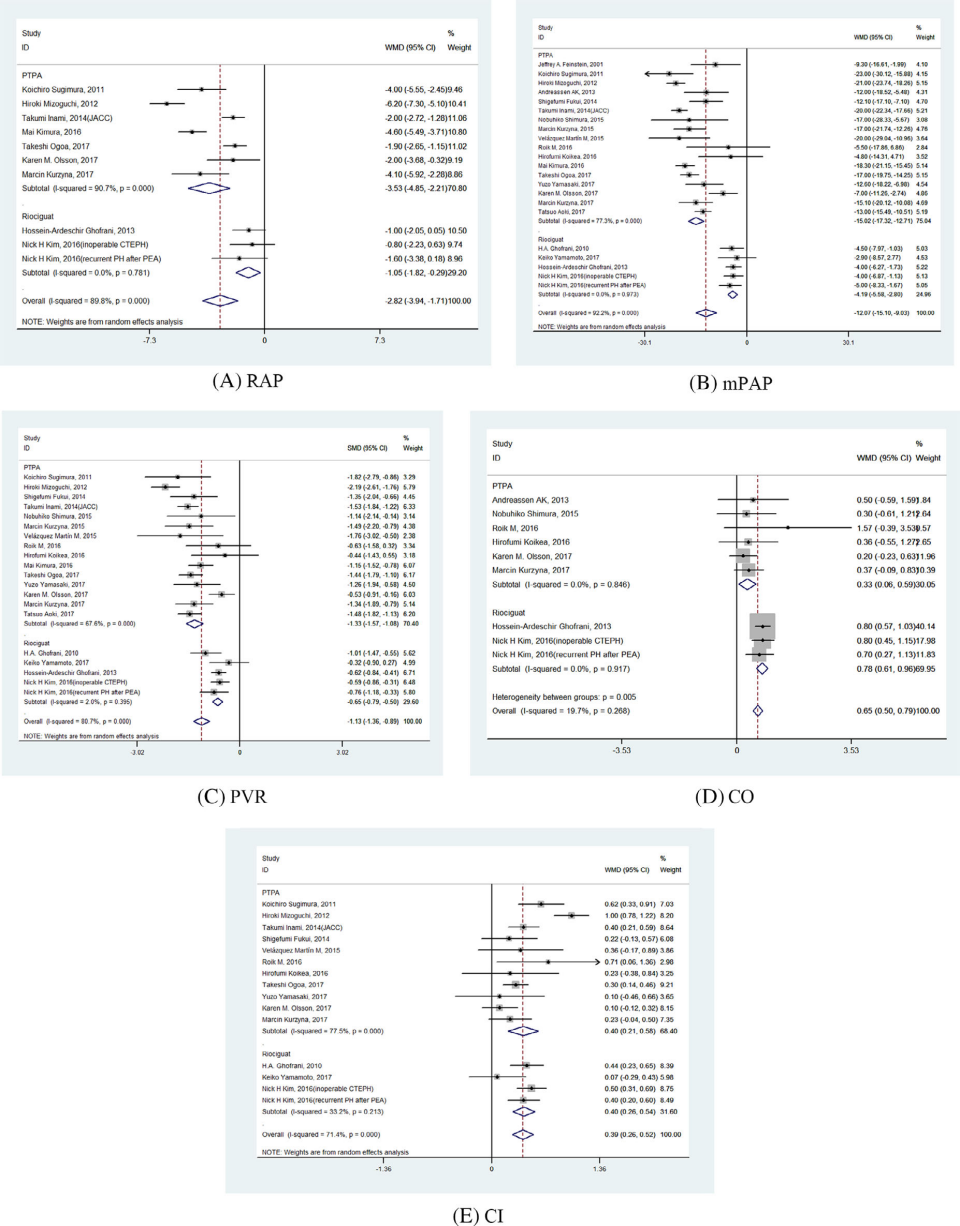# CORRIGENDUM

**DOI:** 10.1002/clc.23330

**Published:** 2019-12-30

**Balloon pulmonary angioplasty vs riociguat in patients with inoperable chronic thromboembolic pulmonary hypertension: A systematic review and meta‐analysis**

Wuwan Wang^1^* | Li Wen^1^* | Zhengdong Song^1^ | Wenhai Shi^2^ | Ke Wang^3^ | Wei Huang^1^ https://orcid.org/0000-0002-2707-2165

^1^Department of Cardiology, The First Affiliated Hospital, Chongqing Medical University, Chongqing, China

^2^Department of Cardiology, The Sixth People's Hospital of Chengdu, Chengdu, China

^3^Institute of Cardiovascular Diseases of People's Liberation of Army (PLA), Xinqiao Hospital, Army Medical University, Chongqing, China

Correspondence: Wei Huang, MD, PhD, Department of Cardiology, The First Affiliated Hospital of Chongqing Medical University, No. 1 Youyi Road, Yuzhong District, Chongqing, 400016, China.

Email: weihuangcq@gmail.com

*Clinical Cardiology*. 2019;42:741‐752.

doi: 10.1002/clc.23212

The forrest plot of CO (Figure 2, panel D) was the same as CI in Figure 2. Figure 2 has now been updated to reflect the correct plots. The corrected figure appears below.

The authors apologize for this error.

**Figure 2:**